# Development of a computer-aided tool for the pattern recognition of facial features in diagnosing Turner syndrome: comparison of diagnostic accuracy with clinical workers

**DOI:** 10.1038/s41598-018-27586-9

**Published:** 2018-06-18

**Authors:** Shi Chen, Zhou-xian Pan, Hui-juan Zhu, Qing Wang, Ji-Jiang Yang, Yi Lei, Jian-qiang Li, Hui Pan

**Affiliations:** 1 Department of Endocrinology, Endocrine Key Laboratory of Ministry of Health, Peking Union Medical College Hospital (PUMCH), Chinese Academy of Medical Sciences & Peking Union Medical College (CAMS & PUMC), Beijing, 100730 China; 2 National Virtual Simulation Laboratory Education Center of Medical Sciences, PUMCH, CAMS & PUMC, Beijing, 100730 China; 3 Eight-year Program of Clinical Medicine, PUMCH, CAMS & PUMC, Beijing, 100730 China; 4 Research Institute of Informaiton and Technology, Tsinghua University, Beijing, 100084 China; 5 Wuxi Research Institute of Applied Technologies, Tsinghua University, Wuxi, 214072 China; 6 School of Software, North University of China, Taiyuan, 030051 China; 7 School of Software Engineering, Beijing University of Technology, Beijing, 100124 China; 8 Department of Education, PUMCH, CAMS & PUMC, Beijing, 100730 China

## Abstract

Technologies applied for the recognition of facial features in diagnosing certain disorders seem to be promising in reducing the medical burden and improve the efficiency. This pilot study aimed to develop a computer-assisted tool for the pattern recognition of facial features for diagnosing Turner syndrome (TS). Photographs of 54 patients with TS and 158 female controls were collected from July 2016 to May 2017. Finally, photographs of 32 patients with TS and 96 age-matched controls were included in the study that were further divided equally into training and testing groups. The process of automatic classification consisted of image preprocessing, facial feature extraction, feature reduction and fusion, automatic classification, and result presentation. A total of 27 physicians and 21 medical students completed a web-based test including the same photographs used in computer testing. After training, the automatic facial classification system for diagnosing TS achieved a 68.8% sensitivity and 87.5% specificity (and a 67.6% average sensitivity and 87.9% average specificity after resampling), which was significantly higher than the average sensitivity (57.4%, P < 0.001) and specificity (75.4%, P < 0.001) of 48 participants, respectively. The accuracy of this system was satisfactory and better than the diagnosis by clinicians. However, the system necessitates further improvement for achieving a high diagnostic accuracy in clinical practice.

## Introduction

Turner syndrome (TS), one of the most common chromosomal disorders, results from the total or partial absence or structural abnormality of one copy of X chromosome. The prevalence of TS in live-born females is approximately 1/2500^1^. Approximately, 50% of the individuals with TS have a complete 45,X monosomy, whereas the remaining 50% show mosaicism^2^: {45,X/46,XX}, {45,X/46,XiXq}, {45,X/46,XY}, and {45,X/46,XrX}^1,3^. The clinical presentations include short stature, ovarian failure secondary to gonadal dysgenesis, webbed neck, lymphedema, cardiovascular anomalies, renal anomalies, sensorineural hearing loss, autoimmune disorders, and other multisystem affections^1,4^. The diagnosis of TS is often delayed as none of the characteristics mentioned above are diagnostic. Thus, a standard 30-cell karyotype analysis must be performed for the clinical diagnosis of TS, which requires a prolonged period. Earlier diagnosis, as well as earlier initiation of treatment, is crucial for the outcome of TS. For example, starting growth-promoting therapy at a young age maximizes the adult stature of TS. Also, the induction of puberty by appropriate hormone therapy is vital for normal appearance of the female and psychosocial adjustment, as well as, the maintenance of bone mineral density^5,6^. Novel diagnostic approaches need to be explored for the early diagnosis of TS. Thus, a cheap, quick, noninvasive, and convenient diagnostic method used for screening the TS in all girls of short stature can be promising for minimizing the delay in the diagnosis of TS.

Artificial intelligence (AI)/machine learning (ML) has been integrated into routine clinical practice, especially for providing diagnostic support such as detection of pulmonary nodules^7^, skin lesions^8^, colonic polyps^9^, mammographic lesions^10,11^, and oral squamous cell carcinoma^12^ or assistance in identifying prognostic factors for cancers^13^. As one of the dominant areas of AI, a technology involving the pattern recognition of facial features has also been incorporated into the assisted diagnosis of genetic syndromes and endocrine disorders with dysmorphic facial patterns. Consequently, facial image analysis software is used for diagnosing Cushing’s syndrome and acromegaly, which is not only diagnostically accurate but also effective in early diagnosis as compared to that by physicians^14–16^. Similarly, the computer accuracy is higher than that by experts in recognizing multiple genetic syndromes, indicating that these technologies are crucial in a clinical setting^17,18^. However, the disease spectrum explored in these studies was not sufficient, and the sample size was small.

Individuals with TS present disease-specific facial malformations, including epicanthus, ptosis, ocular hypertelorism, low-set ears, multiple facial nevi, low hairline, micrognathia, and webbed neck^2,3,19,20^. However, no study is yet published on the application of facial analysis technology for the detection of TS. In this study, an automatic facial classification system was applied for the diagnosis of TS, and the classification accuracies of the system and clinical workers were compared.

## Methods and Subjects

### Criteria for photographs

All the photographs were acquired at the Short Stature Clinic, Department of Endocrinology, Peking Union Medical College Hospital (PUMCH), in a standardized manner: (1) a frontal picture was taken using a regular digital camera (Panasonic DMC-FZ5 digital camera, Panasonic, Osaka, Japan) with participants sitting in front of a blue background; (2) hats and glasses were removed, and long hair was tied to show the ears; and (3) neutral facial expression with eyes straight ahead and mouth was closed.

### Patients and controls

Photographs of 212 female participants (54 patients with TS and 158 controls), Han Chinese origin, were collected from July 2016 to May 2017. The TS was diagnosed by karyotype analysis. One patient with lipoatrophy, one with Cushing’s syndrome, and one with McCune–Albright syndrome were excluded from the control group owing to a significant facial deformity, which might interfere with the outcome of facial classification. The remaining 155 controls included 17 patients diagnosed with growth hormone deficiency, 4 with partial growth hormone deficiency, 7 with small for gestational age, 20 with precocious puberty, 36 with idiopathic short stature, 3 with hypothyroidism, and 68 healthy controls.

**Figure 1:**
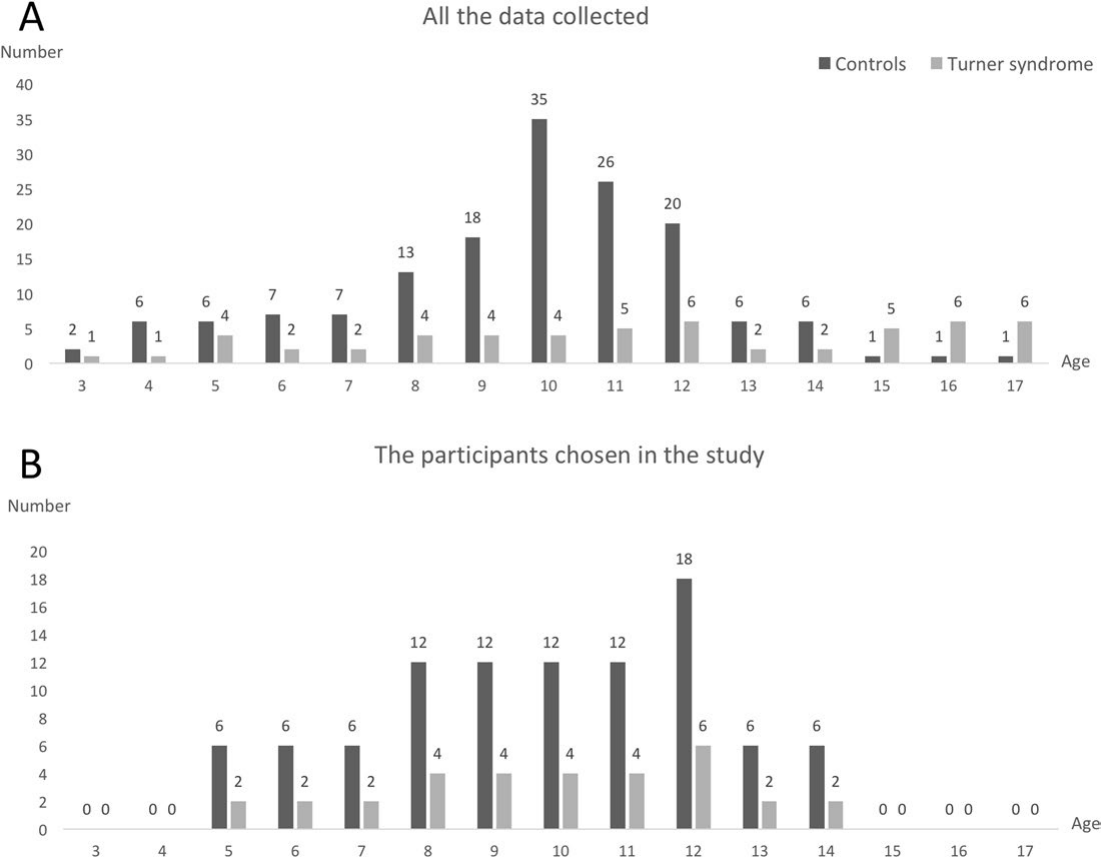
Age distribution of patients and controls. The black bar represents the number of controls. The gray bar represents the number of patients. (**A**) The age distribution of all participants. (**B**) The age distribution of the participants chosen for the study. The ratio of the number of controls to patients was 3:1 in each age group.

**Figure 2:**
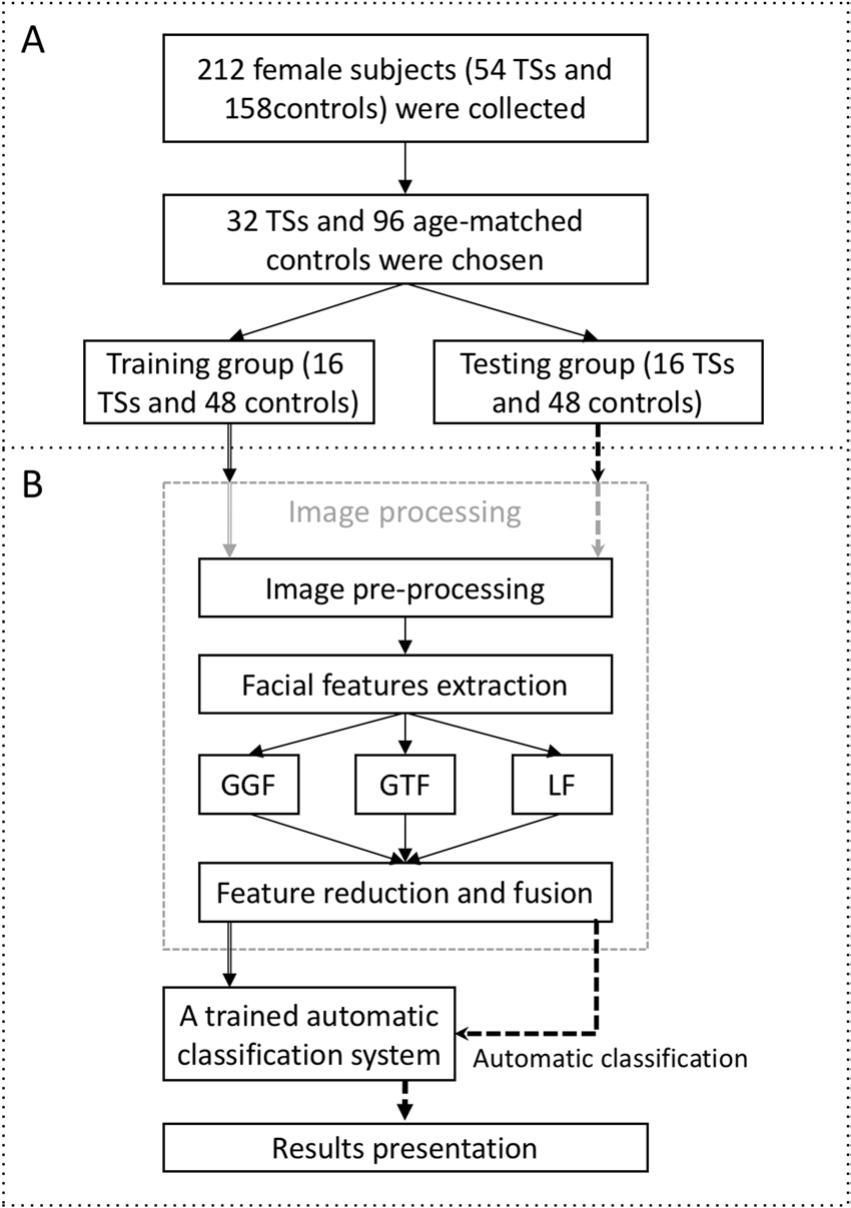
Schematic representation of the study design. (**A**) Collection and selection of photos. (**B**) Framework of automatic facial classification system.

The age distribution of all participants is shown in Fig. 1a. As age was a major determinant of facial appearance, the data needed to be sorted in an age-matched manner. In order to make the most of data, the most appropriate ratio of the number of patients to controls in each group of age was found to be 1:3. The number of patients in each group must be even as they are further divided equally into training and testing groups. Since five patients were present in the 11-year-old group, one patient was excluded from this group to maintain an even number of patients. The age groups of <5-year-old and >15-year-old were excluded owing to the mismatched data between the TS and control groups. Finally, 32 patients with TS and 96 controls were selected (Fig. 1b). Participants in the same age group were divided into training and testing groups evenly and randomly; thus, the testing and training groups comprised of 16 patients with TS and 48 controls (Fig. 2a).

### Face classification by computer

#### The process of automatic classification

The process of automatic classification consisted of five parts (Fig. 2b).

**Image preprocessing:** Each photograph was preprocessed according to the following three steps. (1) Geometric normalization: First, the three characteristic points of the eye and nose were calibrated with the mouse, and the coordinates of the three feature points were obtained. Then, based on the coordinates of the left and right eyes, the image was rotated to ensure the consistency of the direction of the face. Finally, the images were zoomed out to the size of 640 × 640; (2) Face area recognition and interception; (3) Gray-scale normalization: The brightness of the image was increased to clarify the details and reduce the impact of light intensity. Histogram equalization was used for the calibration of the illumination.

**Figure 3:**
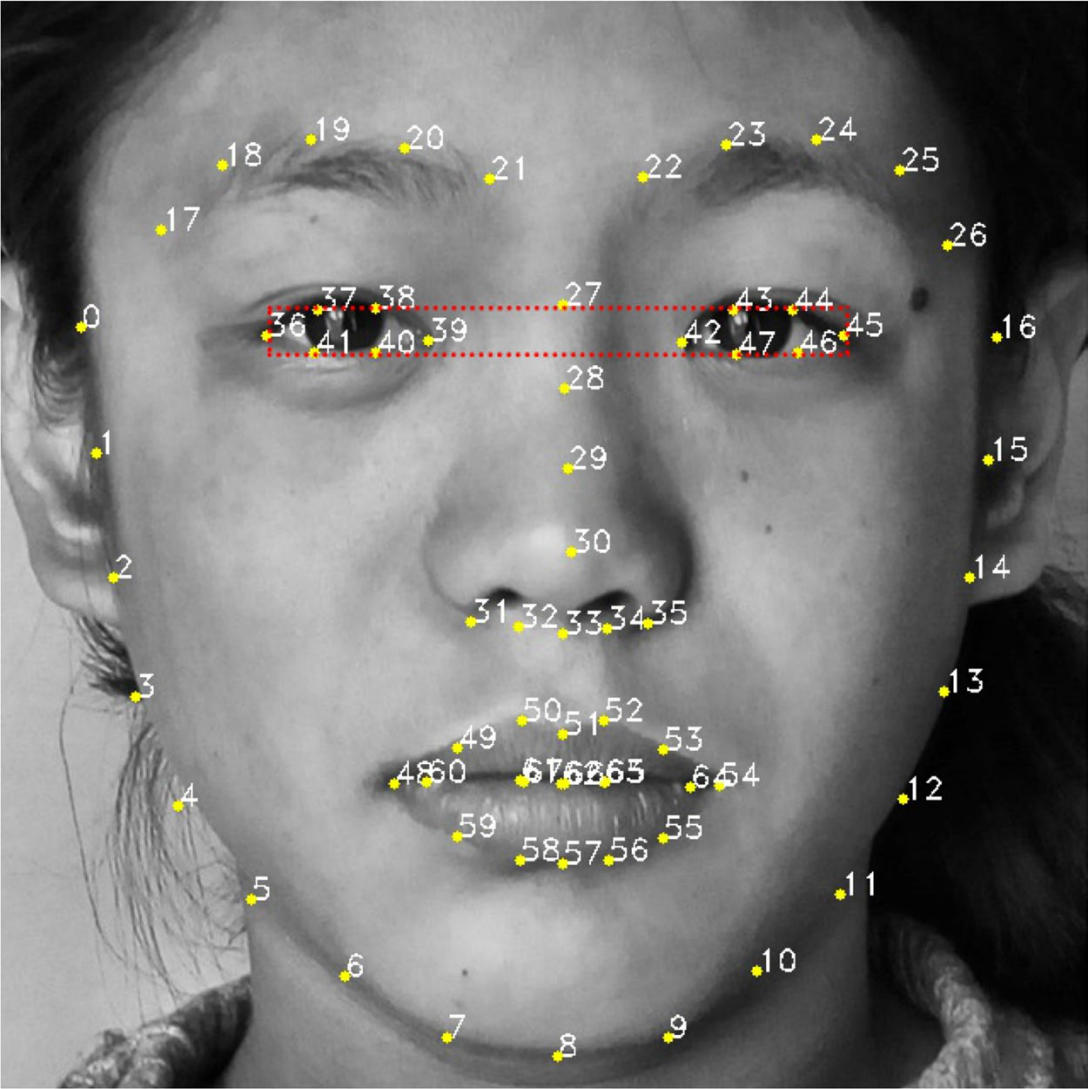
Sixty-eight-feature landmark face model of a patient diagnosed with Turner syndrome.

Subsequently, we trained a 68-feature point face model and segmented the eye region based on 6 points in the 68 points (Fig. 3). The 68-feature points can be detected and tracked using the method proposed by Kazemi *et al*.^21^. The point numbers 0–16 represent the outline of the face, 17–21 and 22–26 represent the area of the right and left eyebrows, respectively, 27–35 represent the area of the nasal bridge, 36–41 and 42–47 represent the area of the right and left eyes, respectively, and 48–67 represent the mouth. Then, suitable geometric and texture features were extracted based on the feature points and the regions defined by the feature points.

**Facial feature extraction:** Global geometrical features (GGF), global texture features (GTF), and multiple local features (LF) were extracted and analyzed on the computer.

GGF: First, a coordinate system was set up on the preprocessed facial image, which retrieved 68 coordinate values as p_*n*_ (n = 0, $\cdots$,67). Second, the Euclidean distance d_*ij*_ (i, j = 0, $\cdots$, 67 i ≠ j) between the two different feature points was calculated^22^. Finally, we obtained 2278 distance features for each facial image.

GTF: GTF was extracted based on Gabor wavelet transformation. The Gabor wavelet transformation is similar to the biological effects of the human eyes. Images were cropped to the size of 128 × 128 in GT feature extraction in order to reduce the computational complexity. Then, we designed the Gabor filter group comprising of 5 scales (1, 2, $\cdots$,5) and 8 (1, 2, $\cdots$,8) directions to extract the facial images’ features of different scales and directions for the construction of the eigenvector. After obtaining the filter groups, each facial image was divided into 8 × 8 blocks, and then, the energy of each block was calculated^23^.

LF: Detailed local features can improve the recognition accuracy. Five LFs (epicanthus, melanocytic nevus, ocular distance, forehand, and nasal bridge) were extracted using three methods (Table 1). The geometrical features of the forehead, nasal bridge, and ocular distance were extracted based on the 68 feature-point model, denoted as V_*L*1_, V_*L*2_, and V_*L*3_, respectively. Then, the Gabor wavelet filter was used for extracting the texture feature of epicanthus in the eye area, which effectively enhanced the feature of epicanthus, denoted as V_*L*4_. Subsequently, a Laplacian of Gaussian filter was used for the detection of melanocytic nevus^24^. The number and size of melanocytic nevi were merged into one vector, denoted as V_*L*5_.

**Table 1:** Extraction method of each local feature.

Feature	Method
Forehead	Calculate the Euclidean distance between point numbers 17 and 26
Multiple facial nevi	Blob detection
Epicanthus	Gabor wavelet filter without dividing blocks
Nasal bridge	Calculate the Euclidean distance between point numbers 30 and 33
Ocular distance	Calculate the ratio between d_39,42_ and d_36,45_

Taken together, a 1 × 2278 matrix by calculating the Euclidean distance between feature landmarks, a 1 × 64 × 64 joint spatial frequency energy matrix of Gabor features, and a fusion matrix of LFs were achieved for each image.

**Figure 4:**
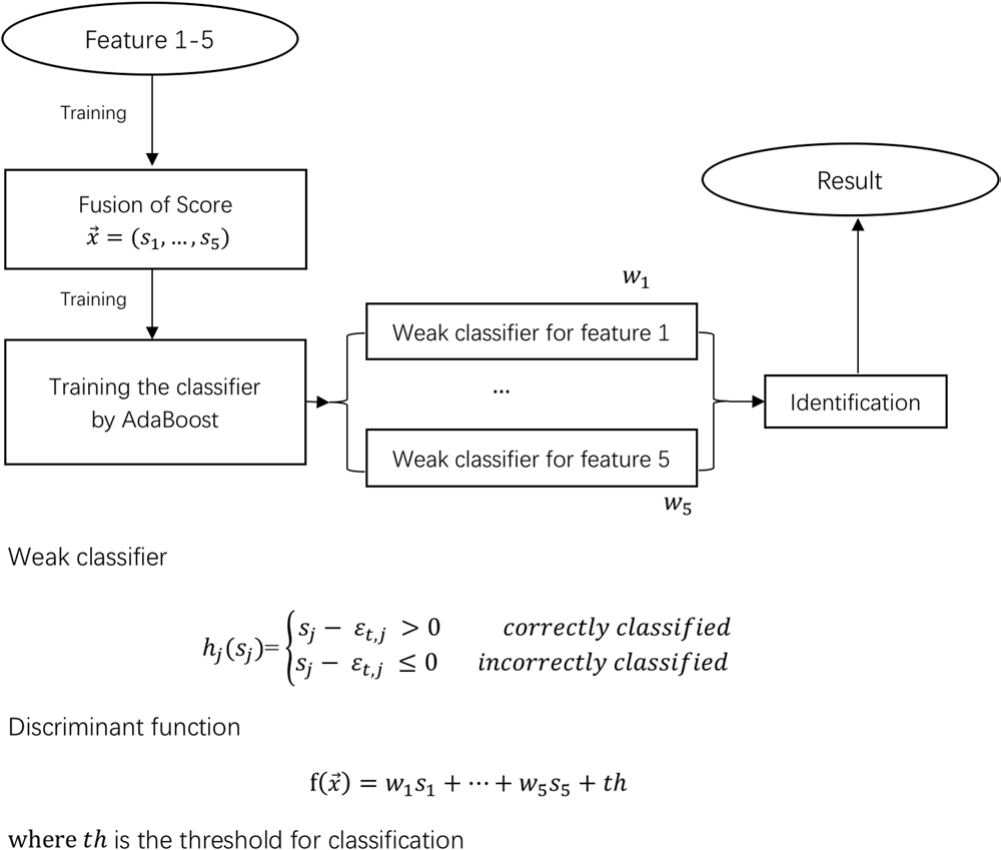
Order of applying AdaBoost in this experiment.

**Feature reduction and fusion:** The principal component analysis (PCA) method was applied to reduce the dimensionality of the data and enhance the discriminating ability^25^. The 2278 dimension of the global texture feature was automatically reduced to 36 dimensions, and the 4096 dimension of the global texture feature was automatically reduced to 24 dimensions. The number of dimensions was the result of automatic calculation of the PCA algorithm, and what each number represented was not explanatory. The AdaBoost algorithm was used for the score-level fusion of the five local facial features^25,26^. Figure 4 shows the functional order when AdaBoost was applied in this experiment.

**Automatic classification:** AdaBoost classifier was used for the classification of the local features. Support vector machine (SVM), a kind of binary classifier with robust learning and generalization abilities harbor unique advantages in solving small sample problems. The SVMs were used for the classification from GGFs and GTFs. For nonlinear and high-dimensional recognition problems, classification of global features was essential due to the small sample size in the current study.

The K-fold (k = 10) cross-validation technique was applied to the training set in order to select the best features and the method to recognize TS. Subsequently, the trained classification system was used for the automatic classification of the participants in the testing group.

**Result presentation:** The results of automatic classification were presented.

#### Random resampling of training and testing groups

As described above, 32 patients with TS and 96 controls in the same age group were divided into training and testing groups evenly and randomly. For robust statistical results, we resampled the training and testing groups, followed by automatic classification that was conducted 50 times, and then, the average sensitivity and specificity were computed.

### Face classification by physicians and medical students

A web test consisting of the same photographs of 16 patients with TS and 48 controls used in computer testing was conducted among the departments of endocrinology, pediatrics, and gynecology in PUMCH and the other three hospitals of different levels. One of the three hospitals, as well as PUMCH, belong to the Ministry of Health in China; one is a provincial hospital and the other a city hospital. A total of 48 participants completed the test voluntarily. None of them visited the short stature clinics of PUMCH. In addition to photographs, the web test contained a theory test with seven “true or false” questions about the facial features of TS: epicanthus, ocular hypertelorism, low-set ears, multiple facial nevi, low hairline, micrognathia, and flat nose. The first 6/7 features constituted the facial features of TS^2,3,19,20^. Each correct answer was awarded 1 point, and thus, the full score of the theory test was 7 points.

### Ethics approval

The ethical approval for the present study was obtained from the Institutional Review Board of Chinese Academy of Medical Sciences, PUMCH (Project No: ZS-1242). All patients provided a written informed consent. The patient in Fig. 3 signed the informed consent for the publication of her facial image in an online open-access publication. The study procedures were performed in accordance with the approved guidelines.

### Statistical analysis

Data were computed and analyzed using IBM SPSS statistical package for Mac, version 23 (IBM Co., NY, USA). The distribution of continuous data was expressed using mean and standard deviation (SD). The one-sample t-test was used to compare the classification accuracies between clinical workers and the computer. One-way analysis of variance (ANOVA) analyzed the comparison of classification accuracies between subgroups of clinical workers after testing for homogeneity. Pearson’s correlation coefficient was used to evaluate the correlation between classification accuracy and the score of theory test. A P-value < 0.05 indicated statistical significance.

## Results

### Participants

A database consisting of 54 photographs of patients with TS and 155 photographs of female controls from July 2016 to May 2017 was constructed. The age distribution of the TS and control groups varied significantly (average age: TS 11.4 vs. control 9.7 years, P < 0.001). A total of 32 girls with TS and 96 age-matched controls were selected randomly to achieve age-matched data. The average age of all participants was 9.8 years (Fig. 1b).

### Classification accuracy of the automatic classification system

The results of automatic classification system using three different methods are presented in Table 2. Of the three methods, an AdaBoost classifier with the extraction of LF exhibited maximal sensitivity (correctly classified 68.8% of the patients as compared to 25% by GGF and 37.5% by GTF) and specificity (correctly classified 87.5% of the controls as compared to 81.3% by GGF and 87.5% by GTF). Furthermore, the results of the AdaBoost classifier system (68.8% and 87.5%) were compared to those of participants.

**Table 2:** Classification accuracy of automatic classification system.

Feature	Method	Performance on the testing set	
		Sensitivity	Specificity
Global geometrical features	PCA + SVM	4/16 = 25%	39/48 = 81.3%
Global texture features	PCA + SVM	6/16 = 37.5%	42/48 = 87.5%
Fusion of local features	AdaBoost	11/16 = 68.8%	42/48 = 87.5%

SVM, support vector machine.

PCA, principal component analysis.

#### Results of random resampling of training and testing groups

After resampling, the automatic classification was run for 50 times, and the average sensitivity and specificity were shown in Table 3. None of the average sensitivities and specificities of resampling were significantly different from those of the previous sampling, except the sensitivity of GGF with SVM.

**Table 3:** Average classification accuracy of the automatic classification system after random resampling.

Feature	Method	Average performance on the testing set			
		Average sensitivity	P-value	Average specificity	P-value
Global geometrical features	PCA + SVM	23.8 ± 15.2%	0.56	80.8 ± 8.1%	0.63
Global texture features	PCA + SVM	44.0 ± 15.7%	0.01*	87.5 ± 5.7%	1.00
Fusion of local features	AdaBoost	67.6 ± 14.5%	0.57	87.9 ± 4.5%	0.47

SVM, support vector machine.

PCA, principal component analysis.

The P-value was used for comparing the results of the specific sampling of participants in Table 2 with the average results of 50 times of resampling, using the t-test. *P < 0.05.

### Classification accuracy of physicians and medical students

**Figure 5:**
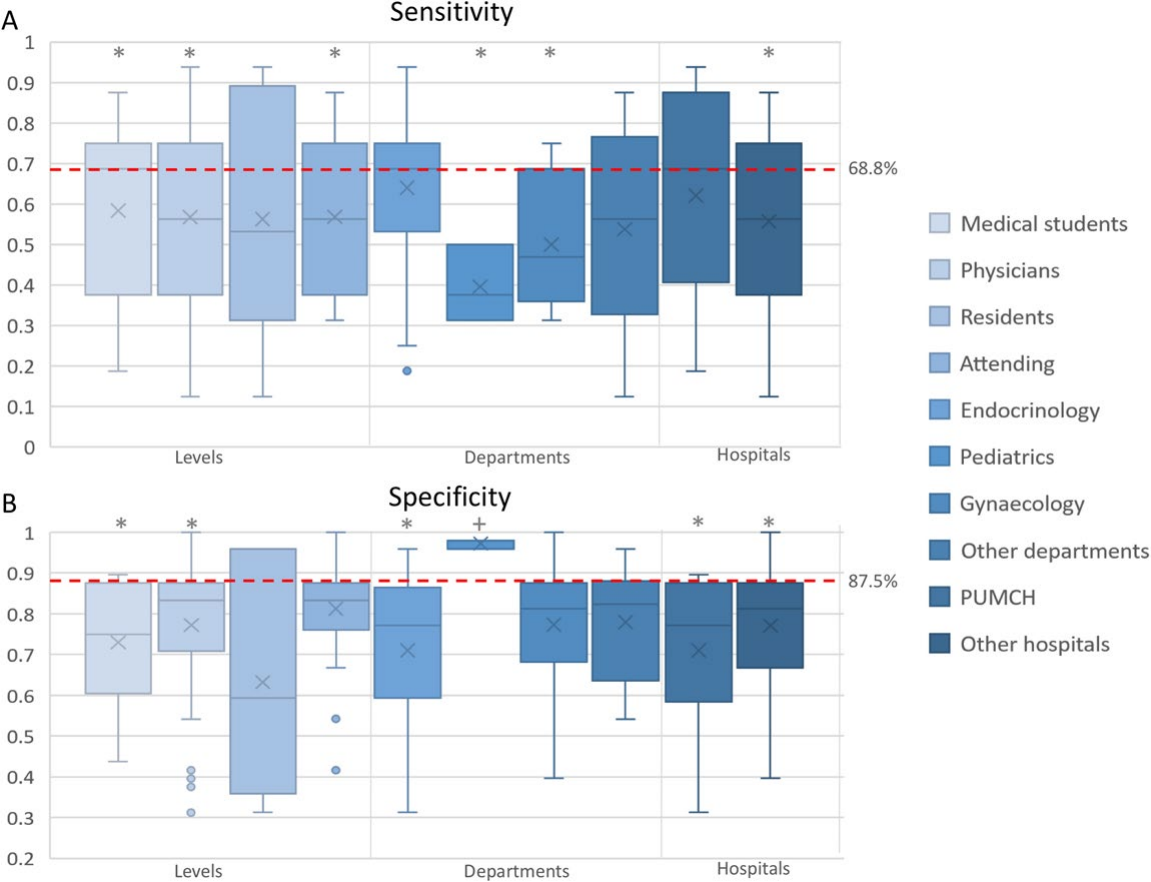
Classification accuracy of doctors and medical students. (**A**) The sensitivity of participants of different levels, different departments, and different hospitals. The dotted line represents the sensitivity (68.8%) of the automatic classification system. (**B**) The specificity of participants of different levels, different departments, and different hospitals. The dotted line represents the specificity (87.5%) of the automatic classification system. *The sensitivity or specificity of the computer was better than that of the participants (evaluated using the t-test; P < 0.05). ^+^The sensitivity or specificity of the participants was better than that of the computer (evaluated using the t-test; P < 0.05).

A total of 48 participants (27 physicians and 21 medical students) completed the web test voluntarily, with an average sensitivity (57.4 ± 21.9%, P = 0.001) and specificity (75.4 ± 17.3%, P = 0.003) significantly lower than that obtained by the computer (Table 4, Fig. 5).

**Table 4:** Classification accuracy of doctors and medical students.

		Classification accuracy			
		Sensitivity(%) (mean ± SD)	P-value	Specificity(%) (mean ± SD)	P-value
All (N = 48)		57.4 ± 21.9	0.001*	75.4 ± 17.3	<0.001*
Levels	Physicians (N = 27)	56.7 ± 21.9	0.01*	77.2 ± 19.8	0.01*
	Attending (N = 21)	56.8 ± 19.2	0.01*	81.3 ± 14.2	0.06
	Residents (N = 6)	56.3 ± 32.1	0.38	63.2 ± 30.4	0.11
	Medical students (N = 21)	58.3 ± 22.4	0.05*	72.6 ± 13.9	<0.001*
Departments	Endocrinology (N = 25)	64.0 ± 20.9	0.27	71.0 ± 17.9	<0.001*
	Pediatrics (N = 3)	39.6 ± 9.5	0.03*	97.2 ± 1.2	0.005^+^
	Gynaecology (N = 10)	50.0 ± 16.9	0.01*	77.3 ± 17.1	0.09
	Others (N = 10)	53.7 ± 27.0	0.11	77.9 ± 14.1	0.06
Hospitals	PUMCH (N = 13)	62.0 ± 25.7	0.36	71.0 ± 19.8	0.01*
	Others (N = 35)	55.7 ± 20.5	0.001*	77.0 ± 16.3	0.001*

SD, standard deviation; PUMCH, Peking Union Medical College Hospital.

The P value was used for comparing the sensitivity or specificity of the participants and computer using the t test.

The sensitivity and specificity of the computer were 68.8% and 87.5%, respectively.

*P < 0.05; worse than the computer. ^+^P < 0.05; better than the computer.

#### Classification accuracy of different levels of physicians and medical students

The sensitivities and specificities of 27 physicians (56.7 ± 21.9%, P = 0.01; 77.2 ± 19.8%, P = 0.01) and 21 medical students (58.3 ± 22.4%, P = 0.05; 72.6 ± 13.9%, P < 0.001) were significantly lower than that of the computers (68.8% and 87.5%, respectively). When the physicians were classified into attending physicians (N = 21) and residents (N = 6), the sensitivity of the attending physicians (56.8 ± 19.2%, P = 0.01) was significantly lower than that of the computer. However, the sensitivity of the residents (56.3 ± 32.1%, P = 0.38) and the specificities of both (81.3 ± 14.2%, P = 0.06; 63.2 ± 30.4%, P = 0.11) were not lower than those of the computer. The One-way ANOVA test did not reveal any significant difference between the sensitivities and specificities of different subgroups (P = 0.97 and 0.05, respectively).

#### Classification accuracy of different departments

Most of the participants majored in endocrinology (N = 25), pediatrics (N = 3), and gynecology (N = 10); the sensitivity, specificity, and P values in comparison with those of computers are shown in Table 4. The One-way ANOVA test did not reveal any significant difference between the sensitivities and specificities of different departments (P = 0.13 and 0.08, respectively).

#### Classification accuracy of different hospitals

A total of 13 participants were from PUMCH. Computer was better than participants from PUMCH in specificity (71.0 ± 19.8%, P = 0.01) and those from other hospitals in sensitivity (55.7 ± 20.5%, P = 0.001) and specificity (77.0 ± 16.3%, P = 0.001), but not better than participants from PUMCH in sensitivity (62.0 ± 25.7%, P = 0.36). One-way ANOVA test did not demonstrate any significant difference between the sensitivities and specificities of different hospitals (P = 0.38 and 0.29, respectively).

### The correlation between classification accuracy and score of the theory test

Pearson’s correlation coefficient of sensitivity and score of theory test was 0.315 (P = 0.03), which showed a slight correlation between the ability of accurate classification of TS and the theoretical knowledge of the facial features of TS. However, the Pearson’s correlation coefficient of specificity and score of the theory test was −0.016 (P = 0.91), which did not establish a correlation between the ability of accurate selection of the control and the theoretical knowledge.

## Discussion

A large number of studies have reported the medical application of AI/ML. The technology involving recognition and classification of facial features in assisted diagnosis is a promising and emerging area of research. The present study developed a computer-assisted tool for the pattern recognition of facial features with high accuracy in the screening and early diagnosis of TS.

The major strengths of this study are described as follows. First, there was no published literature about the use of pattern recognition of facial features in TS. This was the first study to use facial classification in the diagnosis of TS. Second, we compared the accuracy of this system with that of the clinical workers in order to evaluate the utility and efficiency of the classification system. The high accuracy of this system over that of doctors in identifying TS indicates a potential diagnostic method to be used in screening that can avoid a delayed diagnosis of TS. Third, several methods of facial features extraction were used, and attending doctors, residents, and medical students from different hospitals and departments were recruited for a substantial comparison between computers and clinical workers. Fourth, we used stringent experimental conditions: the participants were age-matched, the photographs were uniformly acquired, and the training and testing groups were resampled for 50 times for a robust statistical result.

The automatic face classification system for TS used in this study, with the LF+AdaBoost method, achieved a sensitivity of 68.8% and a specificity of 83.3%, which were better than the diagnostic accuracy of physicians and medical students. The software used for recognizing the facial features in other studies also showed advantages over physicians in specific diseases, such as acromegaly, Cushing’s syndrome, and Cornelia de Lange syndrome^14–17^. This feature highlighted the possibility of the clinical application of computer-assisted recognition of facial features in diagnosing TS in the future. The reason that LF+AdaBoost method had advantages over the other two methods might be attributed to the failure of PCA in preserving the non-linear structure of data during dimension reduction, and that the five local features are representative of the facial features of TS.

However, the sensitivity achieved by a computer (slightly <70%) was not perfect for diagnosing TS; this phenomenon could be attributed to the small sample size in the present study. The patients with TS and controls in the training and testing groups were not height-, weight-, or karyotype-matched due to small sample size, leading to a potential bias in the classification analysis. In addition, the number of participants in each age group was limited and did not fulfill the requirement of a “large training database” to achieve high accuracy. Thus, a sample size in future studies is imperative to improve the diagnostic accuracy of TS.

Although physicians failed to perform better than a computer, it was surprising that no significant difference existed between the recognition accuracies of attending doctors, residents, and medical students. This was inconsistent with the present hypothesis that higher level physicians performed better than those of lower level. A possible reason was that some physicians were confused with respect to TS facial features, which was revealed by the positive correlation between the theory test score and sensitivity. In addition, several patients with TS showed only slight facial changes due to mosaicism, and many facial features of TS (for example, epicanthus, ocular hypertelorism, and multiple facial nevi) were also present in a subset of the normal population, putatively leading to confusion during the classification by physicians and students. Only individuals with a 45, X karyotype tended to have more clinical features as compared to those who were mosaic with a normal cell line^19^. Furthermore, the algorithms used by computers might find differences between patients with TS and controls, which could not be described easily, and thus, were not recorded in the literature.

Nevertheless, the present study had several other limitations in addition to the mismatch of weight, height, and karyotype. First, the age range of participants was 5–14 years. Therefore, this system could not be used for the early detection of patients with TS aged <5 years. Second, not all controls underwent a standard 30-cell karyotype analysis for the exclusion of the diagnosis of TS. Thus, a few children of short stature in the control group might have TS but were not diagnosed. However, this proportion must be extremely small as none of them exhibited any typical features of TS, and the prevalence of the disease was low. Third, a proportion of controls had certain diseases that resulted in short stature, such as growth hormone deficiency and precocious puberty, which might putatively interfere with the results.

## Conclusions

This novel study applied a computer-assisted tool for the recognition of facial features for diagnosing TS. The overall recognition rate of the face classification system was satisfactory and better than the diagnosis by clinicians. Further studies with height-, weight-, and karyotype-matched participants might be promising for improving the diagnostic accuracy in order to eventually generate an automatic face classification system for clinical practice.
